# QTL analysis of soft scald in two apple populations

**DOI:** 10.1038/hortres.2016.43

**Published:** 2016-09-14

**Authors:** Kendra A McClure, Kyle M Gardner, Peter MA Toivonen, Cheryl R Hampson, Jun Song, Charles F Forney, John DeLong, Istvan Rajcan, Sean Myles

**Affiliations:** 1 Department of Plant and Animal Sciences, Faculty of Agriculture, Dalhousie University, Truro, Nova Scotia B2N 5E3, Canada; 2 Department of Plant Agriculture, Crop Science Building, University of Guelph, Guelph, Ontario N1G 2W1, Canada; 3 Agriculture and Agri-Food Canada, Fredericton Research and Development Centre, Fredericton, New Brunswick E3B 4Z7, Canada; 4 Agriculture and Agri-Food Canada, Summerland Research and Development Centre, Summerland, British Columbia V0H 1Z0, Canada; 5 Agriculture and Agri-Food Canada, Kentville Research and Development Centre, Kentville, Nova Scotia, Canada B4N 1J5

## Abstract

The apple (*Malus*×*domestica* Borkh.) is one of the world’s most widely grown and valuable fruit crops. With demand for apples year round, storability has emerged as an important consideration for apple breeding programs. Soft scald is a cold storage-related disorder that results in sunken, darkened tissue on the fruit surface. Apple breeders are keen to generate new cultivars that do not suffer from soft scald and can thus be marketed year round. Traditional breeding approaches are protracted and labor intensive, and therefore marker-assisted selection (MAS) is a valuable tool for breeders. To advance MAS for storage disorders in apple, we used genotyping-by-sequencing (GBS) to generate high-density genetic maps in two F_1_ apple populations, which were then used for quantitative trait locus (QTL) mapping of soft scald. In total, 900 million DNA sequence reads were generated, but after several data filtering steps, only 2% of reads were ultimately used to create two genetic maps that included 1918 and 2818 single-nucleotide polymorphisms. Two QTL associated with soft scald were identified in one of the bi-parental populations originating from parent 11W-12-11, an advanced breeding line. This study demonstrates the utility of next-generation DNA sequencing technologies for QTL mapping in F_1_ populations, and provides a basis for the advancement of MAS to improve storability of apples.

## Introduction

Apples (*Malus×domestica* Borkh.) represent a major horticultural crop internationally, with the second highest production value for a fruit crop.^1^ Although the development of new cultivars focuses mainly on combining fresh fruit quality with pest and disease resistance,^2^ low-temperature-related disorders are also a breeding target as new cultivars are expected to retain their desirable qualities during extended periods of cold storage.^3^ Some cultivars of apples, such as McIntosh, have been grown for well over 200 years and there is an inherent risk with this practice: the pests and diseases that plague a cultivar continue to evolve and change, while a cultivar remains frozen in evolutionary time as it is continually vegetatively propagated.^4^ Confounding this issue is the fact that breeding new cultivars can take substantial time and resources; for example, nearly 30 years passed between the initial cross that generated Honeycrisp and its release as a commercial cultivar.^5,6^

Soft scald, or ribbon scald, is a disorder that manifests in fruit stored ⩽2.2 °C,^7^ after 6–12 weeks storage,^8^ and is thought to result from low-temperature injury.^9^ First described in 1917, its symptoms are a smooth, but sharply defined darkening of the skin, and often the fruit tissue directly below,^7,10^ with patches that vary in size from ⩽0.64 cm diameter to the majority of the skin surface.^11^ Apple cultivars vary in their susceptibility to soft scald, and some high-value cultivars (for example, Honeycrisp) can experience substantial fruit losses due to this disorder.^12,13^ Many factors have been implicated in the severity of soft scald, such as maturity at harvest, growing season conditions, storage settings, fertilizer treatments, rootstock, crop load and soil fertility.^7,12,14^ It is likely that not simply one, but a combination of factors affect soft scald.^12^

Little is known about the biochemistry of soft scald development,^15^ but early work found that it could be induced by injecting apples with either hexanol or hexyl acetate,^16^ while another study found that hexanol, hexanal, hexyl acetate and hexyl butyrate all induced soft scald, with hexanol having the most drastic effect.^17^ In addition, unaffected and affected tissue from the same fruit differed with respect to fatty acid composition: sound tissue had a higher percentage of linoleic acid.^8^ Fatty acid composition also differed between fruit, trees and orchards, suggesting a link to differences in disorder susceptibility.^8^ Despite challenges in determining the underlying cause of the disorder, years of research have found that storing fruit at warmer temperatures, such as 2.2 °C, helps decrease scald in susceptible cultivars.^14^ Other means of reducing soft scald include treatment with the antioxidant diphenylamine (DPA),^15^ warming periods before cold storage,^7,13,18^ and treatment with 1-methylcyclopropene (1-MCP).^19,20^

Although numerous efforts have been made to reduce soft scald incidence in susceptible cultivars, Volz *et al.*^21^ suggest that this disorder is highly heritable and it may, therefore, be possible to breed new cultivars that are resistant to scald. One cost-effective method of developing new apple cultivars is marker-assisted selection (MAS), where offspring are selected at the seedling stage using molecular markers associated with traits of interest.^22^ Apples are an ideal candidate for MAS due to their long juvenile period, large plant size and the expense associated with orchard maintenance.^4^ A marker for soft scald would be especially useful as the disorder may not be expressed under the testing conditions before cultivar release, as was the case for Honeycrisp.^12^ Compared with crops of similar value, few quantitative trait locus (QTL) analyses have been completed in apple.^23^ However, significant research has focused on locating and annotating genes associated with apple scab (*Venturia inaequalis*) resistance.^2^ Indeed, for the most part in apple, implementation of MAS in breeding programs has focused on disease resistance^24^ and some breeding programs are using MAS for monogenic traits such as apple scab resistance.^22^

The release of an annotated apple reference genome^25^ has been essential in the pursuit of genetic studies in apple,^23^ especially for marker discovery using next-generation sequencing (NGS) technology. Generally, increasing the number of markers in a study increases confidence that a trait linked to a true QTL has been discovered, and could be of use for MAS.^24^ Genotyping-by-sequencing (GBS) is a NGS technique that can be used to generate markers for a variety of genetic analyses.^26^ It reduces genome complexity via restriction fragment digestion of isolated DNA, and allows for sample pooling and reaction multiplexing due to barcoded adapter sequences unique to each DNA sample.^26^ The goal of this study was to explore the genetic basis of soft scald using the pseudo-testcross method^27^ for linkage map construction followed by QTL mapping. The fruit from two F_1_ crosses from the Agriculture and Agri-Food Canada (AAFC) Summerland Research and Development Centre apple breeding program were analyzed after cold storage and QTL analyses were performed using single-nucleotide polymorphism (SNP) markers generated using GBS.

## Materials and methods

### Plant material

The plant material used for this study originated from two F_1_ crosses planted at the AAFC Summerland Research and Development Centre in Summerland, British Columbia, Canada that exhibited segregation for soft scald according to the breeder, CRH. Crosses were made between 11W-12-11 (female parent) and SPA440 (male parent), and between Ambrosia (female parent) and Honeycrisp (male parent). Both 11W-12-11 (Summerred×Discovery) and SPA440^28^ (Splendour×Gala) are advanced selections from the Summerland Research and Development Centre apple breeding program. Ambrosia was a chance seedling discovered by Wilfred and Robert Mennell of Cawston, BC, Canada in the 1980s,^29^ and its parentage is unknown. Honeycrisp was developed by the University of Minnesota and was originally thought to be a cross between Macoun×Honeygold,^6^ but this parentage was later found to be incorrect. Honeycrisp is now believed to be a cross between Keepsake and an unknown parent.^30^

For both crosses, emasculated female flowers were pollinated using dried pollen from the male parent at the balloon flower stage. After harvest, seeds were extracted and stratified for 2.5–3 months in sterile sand. The following spring, germinated seeds were planted in styrofoam plug trays in the greenhouse for 1 month before being transplanted to the seedling nursery and grown for 2.5 years. The 11W-12-11×SPA440 cross was made in 2001 and 2003, while the Ambrosia×Honeycrisp cross was made in 2006. Trees were double budded in August onto either Budagovsky 9 (B.9) (11W-12-11×SPA440) or Malling 9 (M.9) (Ambrosia×Honeycrisp) rootstocks and trained as short spindles. The trees were planted in double rows, with 0.6 m between trees within a row, and alternating 1.8 and 3.0 m between rows. All pest management techniques followed industry standards. Trees were drip irrigated and a weed-free strip was maintained under the trees. Crop load was adjusted on all trees by hand thinning to one fruit per cluster with 10–15 cm between each fruit. Hand thinning was completed by early July each year.

### Phenotyping

Disease incidence was measured on trees bearing a minimum of 20 fruit. Beginning in the last week of August, fruit maturity was assessed twice weekly using a delta absorbance (DA) spectrometer (Sinteleia, Bologna, Italy). The DA meter has emerged as a potential non-destructive tool for assessing apple maturity, via measurement of chlorophyll *a* content in apple peel.^31^ More specifically, the DA meter generates a measure of chlorophyll content by calculating the absolute difference in absorbance maxima of two wavelengths of light.^31^ Trees with fruit index of absorbance difference (*I*_AD_) measurements ⩽0.7 were flagged and five fruits per tree were measured again later that week. Once the mean fruit *I*_AD_ value fell below 0.5, the tree was harvested.^32,33^ Fruits were placed in labeled craft paper bags and kept in refrigerated air (RA) storage at 0.5 °C for 3 months at the Summerland Research and Development Centre. After storage, fruit were visually assessed for soft scald presence and severity by noting the hallmarks of disorder manifestation (that is, smooth, sharply defined and darkened skin).^7^ For QTL mapping, scald phenotype measurements were converted to percentage of fruit in a sample that exhibited scald (that is, incidence of disorder). Mapping was also conducted with data converted to binary format (that is, presence/absence of scald).

### Genotyping

Leaf tissue was collected using a hole punch from each accession in each cross once, and collected in duplicate from both sets of parents. Punched tissue was dried using a freeze dryer before being shipped to the AAFC Kentville Research and Development Centre in Kentville, Nova Scotia, Canada for DNA extraction. Tissue was lyophilized and then ground using a 2010 Geno/Grinder (SPEX SamplePrep, Metuchen, NJ, USA). Whole-genome DNA was extracted using a NucleoSpin 96 Plant II kit (Machery-Nagel, Düren, Germany) with the following modifications to the kit protocol: samples were incubated in lysis buffer for 60 min, and were processed using a vacuum manifold with an additional step of filtering lysate through receiver plates before proceeding to the binding step. DNA samples were quantified using the QuantiFluor dsDNA System and the GloMax-Multi+Microplate Multimode Reader with Instinct (Promega, Madison, WI, USA).

Library preparation and DNA sequencing were performed at L’Institut de Biologie Intégrative et des Systèmes (IBIS) at Université Laval, Quebec City, Québec, Canada using an Illumina HiSeq 2000 and the GBS approach,^26^ with the restriction enzyme *Ape*KI.^34^ Each F_1_ progeny was sequenced once, while each parent in each cross was sequenced in duplicate as recommended by Gardner *et al*.^35^

### SNP calling

A custom python script was used to parse the raw sequence file in .fastq format by unique barcode identifiers and to complete several quality-filtration steps.^35^ Any reads missing a barcode and/or restriction fragment tag were removed, and chimeric sequences and adapter sequences were trimmed. The reads from duplicate parent samples were pooled, and seven accessions that produced very low total read numbers were discarded from further analyses. Samples were aligned to version 1.0p of the apple reference genome (https://www.rosaceae.org/species/malus/malus_x_domestica/genome_v1.0p) using bwa^36^ allowing 4% sequence mismatch. Samples were grouped by population (11W-12-11×SPA440 and Ambrosia×Honeycrisp), and SNPs were called using the UnifiedGenotyper tool in GATK.^37^ After calling SNPs, filtering was done using VCFtools^38^ with a maximum of 20% missing data per SNP, a minor allele frequency threshold of 0.20, and a minimum read depth of 8. SNPs were grouped for pseudo-testcross consensus map construction into three groups and checked for segregation distortion using a chi-squared test: (i) markers heterozygous in parent 1 and homozygous in parent 2 (Aa×aa), (ii) markers homozygous in parent 1 and heterozygous in parent 2 (aa×Aa) and (iii) markers heterozygous in both parents (Aa×Aa). For further SNP calling details, see the original protocol.^35^

### Consensus map construction

SNPs were binned in each population by 2 cM windows and those from unassembled regions of the genome were removed before consensus map construction. A CP type (outbreeder full-sib family) consensus map was constructed for each population using JoinMap 4.0,^39^ with *N*=221 for the 11W-12-11×SPA440 cross, and *N*=119 for the Ambrosia×Honeycrisp cross. Markers were placed into linkage groups using the independence log10 likelihood ratio (LOD) option, and ordered on groups using the regression mapping algorithm and the Kosambi mapping function. The start order option was used, where markers were first ordered in one parental background using R/qtl and then fed into JoinMap. Any markers that had NNFIT values ⩾50 were removed. Consensus maps for each cross were drawn using MapChart.^40^

### QTL analysis

Interval mapping was conducted using R/qtl version 1.36–6,^41^ using the scanone function. In 2013, phenotype data were available for 213 progeny of the 11W-12-11×SPA440 cross, and 91 progeny of the Ambrosia×Honeycrisp cross (Supplementary Figure S1). In 2014, phenotype data were available for 71 progeny in each cross (142 phenotype data points total) due to inadequate crop load (Supplementary Figure S1). QTL analyses were repeated, but no significant QTL were detected from the 2014 data (data not shown). The model=’binary’ option of scanone was used to compare interval mapping of phenotype data as untransformed or binary (0=0–15% incidence, 1=16–100% incidence). Problematic phasing of markers was highlighted by plotting the pairwise recombination fractions and LOD scores using the plotRF function, and switched using the switchAlleles function before consensus map construction and subsequent QTL analysis. A 5% LOD significance threshold was generated for each QTL analysis using 10 000 permutation tests. The proportion of variance explained by a QTL peak was calculated using 1–10^−2LOD/*n*^, where *n*=sample size.^42^ An interval of ±50 kbp around SNPs with the highest LOD score on each chromosome was searched for genes using the GBrowse tool for *Malus*×*domestica* v1.0 pseudo haplotype (https://www.rosaceae.org/gb/gbrowse/malus_x_domestica_v1.0-primary/). The PLINK genotype files, JoinMap input files, and phenotype data will be made available through the Dryad Digital Repository (http://datadryad.org).

## Results

### SNP generation and filtering

**Figure 1:**
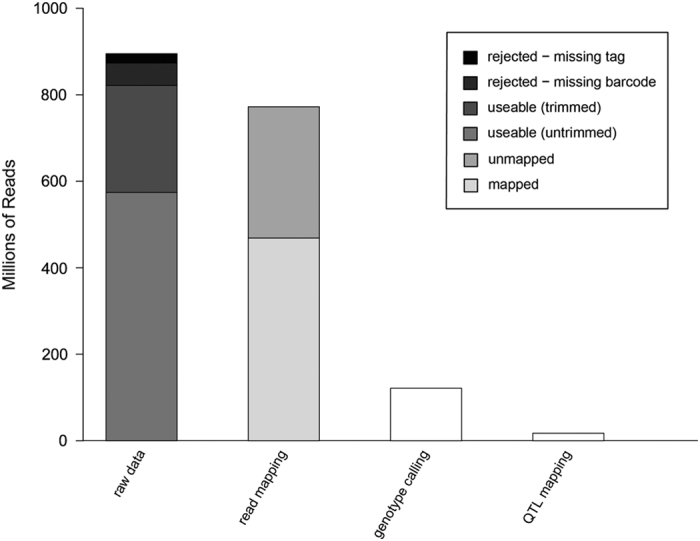
Number of reads across quality-filtering steps. A total of 894 948 266 reads were generated. In the first step (raw data), reads were rejected if they lacked a restriction fragment tag or a barcode; and were trimmed if they were chimeric or contained an adapter sequence. After these filters were applied, seven samples with low read counts were removed, further decreasing read counts. In the next step (read mapping), reads were rejected if they did not uniquely map to the reference genome. The number of reads was then reduced to the next bar (genotype calling) by filtering for minor allele frequency (MAF), read depth and data missingness. Markers unanchored to the 17 chromosomes of the apple genome were also removed. Finally, in the final bar (QTL mapping), SNPs were retained only if they passed filters for segregation distortion and marker duplication. The final set of SNPs for QTL analysis were derived from 17 395 037 reads, or about 2% of the original sequence data.

The first step following sequencing was to process the raw DNA sequence data through a custom read filtration pipeline.^35^ Read counts were fairly consistent across all four plates sequenced; each plate of 96 samples generating >200 million reads (Figure 1, ‘raw data’). Seven samples failed to generate a sufficient number of reads and were discarded from further analyses. A total of 73 544 759 reads were also discarded for missing a barcode or restriction fragment tag. Finally, 821 403 507 reads that were deemed ‘good reads’ for downstream analyses also included those that were trimmed because they were adjacent to chimeric restriction fragment regions, or reads that read into a sequencing adapter region. Of the remaining reads, 39% were discarded because they did not map uniquely to the reference genome (Figure 1, ‘read mapping’). The remaining reads were then used to call SNPs using GATK. Additional stringent filtration steps included filters implemented in VCFtools (Figure 1, ‘genotype calling’), and this resulted in 26% of the total mapped reads being retained. At this point, markers that did not map to any of the 17 apple chromosomes—‘unassembled’ markers—were also removed from downstream analyses.

### Consensus map construction

All SNPs that passed VCFtools thresholds were divided into the three marker state categories for the pseudo-testcross linkage map construction. Markers were filtered for Mendelian segregation and binned to ease linkage map construction. What remained were 1918 markers for the 11W-12-11×SPA440 (*N*=221) consensus map (Supplementary Figure S2), and 2818 for the Ambrosia×Honeycrisp (*N*=119) map (Supplementary Figure S3). For the final QTL analysis for each parental background ([Aa×aa] and [aa×Aa]), 1355 markers were used in the 11W-12-11×SPA440 population (*N*=213 with phenotype data in 2013), and 1838 in the Ambrosia×Honeycrisp population (*N*=91 with phenotype data in 2013). These sets of SNPs were generated from 9 771 701 and 7 623 336 reads, respectively (Figure 1, ‘QTL mapping’). In total, of the nearly 900 million reads generated, 17 395 037, or 2%, were used for the final QTL analyses.

It was noted that parent SPA440 had substantially fewer reads compared to parent 11W-12-11, which had >7 million more reads. In the Ambrosia×Honeycrisp cross, the parents’ read counts were more evenly distributed, differing by <1 million. The parental maps of the Ambrosia×Honeycrisp cross were well saturated with markers for each linkage group. SPA440 had few markers on chromosome 1, and not enough markers grouped to chromosome 5, hence, it is absent in Supplementary Figure S4. Few markers also grouped to chromosome 6 in 11W-12-11. Preliminary QTL analyses in R/qtl with all markers before linkage mapping suggested that loss of markers on these linkage groups did not drastically affect QTL analysis results.

The consensus map for 11W-12-11×SPA440 consisted of 1918 SNP markers, and spanned 1586 cM. The average distance between SNPs across chromosomes was 0.83 cM. For the Ambrosia×Honeycrisp cross, the consensus map contained 900 more markers (2818), and was 1464 cM in length. The average distance between markers across chromosomes was 0.52 cM.

### QTL analysis

**Figure 2:**
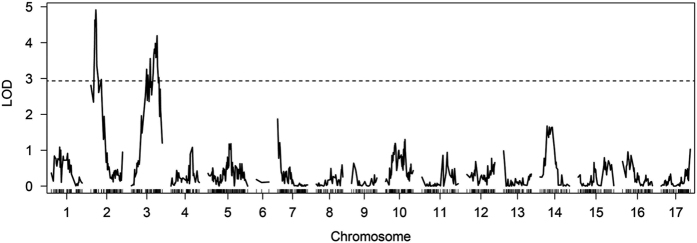
QTL analysis results for parent 11W-12-11 across the 17 apple chromosomes. The solid line represents LOD scores for the QTL analysis, while the dotted line represents the significance threshold based on 10 000 permutation tests. Regions on chromosomes 2 and 3 are significantly associated with incidence of soft scald.

To the best of our knowledge, only one other unpublished attempt has been made to genetically map apple soft scald to date. Significant soft scald QTL were observed for parent 11W-12-11 on chromosomes 2 and 3 using 2013 data (Figure 2). No other significant QTL were detected in the other parental backgrounds (Supplementary Figures S4, S5, S6). The two significant QTL detected from the 2013 data did not show stability across years: no significant QTL were detected in 11W-12-11 from the 2014 data. The correlation in soft scald incidence between years was significant, but relatively weak in both populations (*R*^2^=0.121, *P*=0.00251 in 11W-12-11×SPA440; *R*^2^=0.224, *P*=0.000344 in Ambrosia×Honeycrisp; Supplementary Figure S1).

Scald incidence was skewed towards zero: F_1_ trees often produced fruit that exhibited no symptoms of scald (Supplementary Figure S1). No data transformation improved normality, therefore the model=‘binary’ option in scanone was used to see if mapping the data as a binary trait drastically changed results, which it did not (Supplementary Figure S7). As a result, all further analyses were completed with the data in its original form.

**Figure 3:**
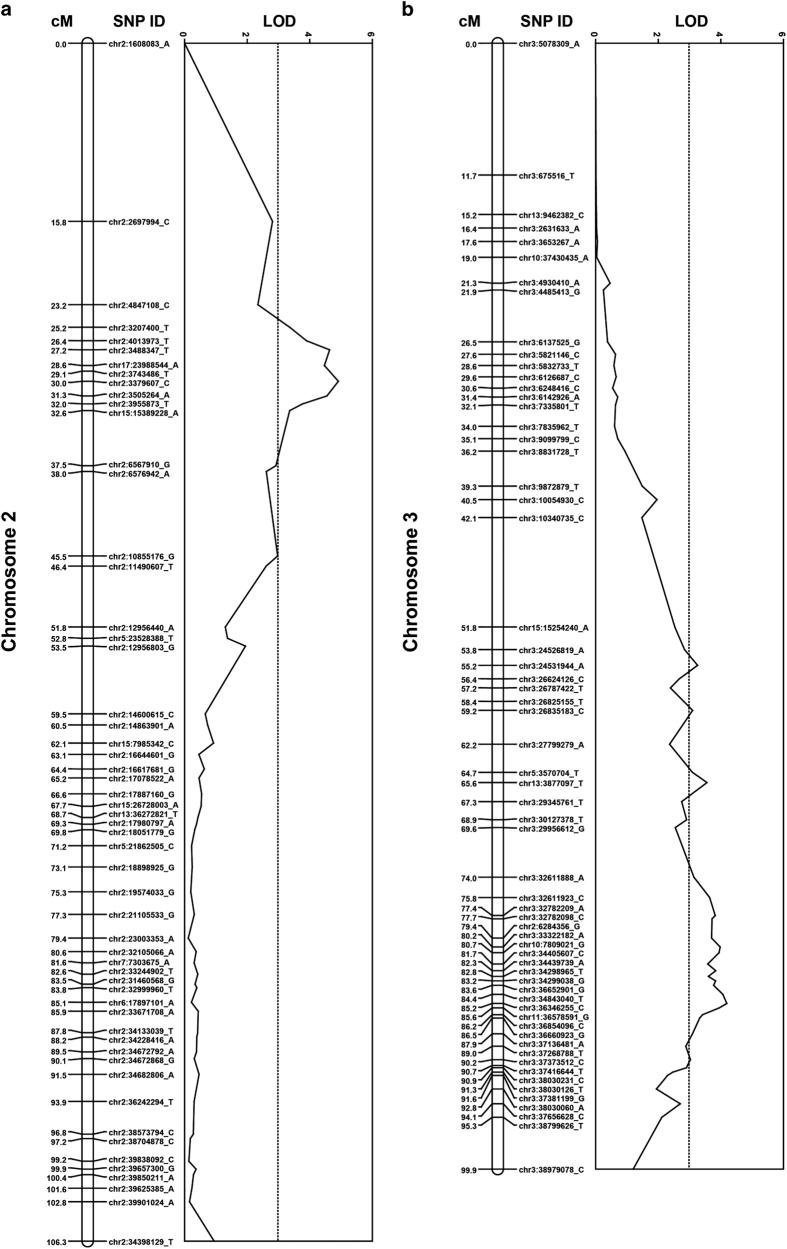
Soft scald QTL analysis results for parent 11W-12-11. LOD scores for QTL analysis results for chromosome 2 (**a**) and chromosome 3 (**b**) are denoted by the solid line. The dotted line represents the significance threshold resulting from 10 000 permutation tests. All SNP names reflect their positions according to the Golden Delicious v1.0 reference genome. The highest LOD score for chromosome 2 was marker chr2:3379607_C (4.91), and for chromosome 3 was marker chr3:36346255_C (4.19).

The most significant QTL was obtained on chromosome 2 in 2013 for the 11W-12-11 parental background (Figure 3a). Marker chr2:3379607_C had a LOD score of 4.91, and the proportion of variation explained by this QTL was 10%. Another marker on chromosome 3 (chr3:36346255_C; Figure 3b), had a LOD score of 4.19 and explained 8.7% of variation. When the soft scald values for both marker states at each of these loci were plotted, in both instances the heterozygous state revealed an increase in soft scald values (Supplementary Figure S8). A QTL confidence interval of 100 kbp (±50 kbp) around each peak was selected and these regions of the genome were analyzed using the GBrowse tool of the Genome Database for Rosaceae. For the peak on chromosome 2, no candidate genes were identified, but the predominant GO terms were associated with enzymatic activity or binding (Supplementary Table S1). For the peak on chromosome 3, fewer genes were found within the mapping interval, many of which also had enzymatic activity or binding GO terms (Supplementary Table S2).

## Discussion

Soft scald is a brown-to-black discoloration of apple skin and flesh,^11^ as a result of low temperature or chilling injury,^19^ when fruit is stored below 2–3 °C.^15^ Soft scald is a common storage disorder in the cultivar Honeycrisp in Canada,^18^ which should be a concern due to the extensive recent plantings of Honeycrisp.^12^ Although holding Honeycrisp fruit at warmer temperatures before storage helps mitigate soft scald, this pre-storage treatment can negatively affect quality in other cultivars.^15^ Despite its benefits, it is still unclear why a warming period helps prevent soft scald.^13^ Honeycrisp did not display soft scald symptoms during its selection in a breeding program in Minnesota.^12^ Therefore, a marker for soft scald would be of use for the development of new apple cultivars to avoid the storage problem Honeycrisp growers now face. To the best of our knowledge, little research has explored the genetic basis of soft scald, but Volz *et al.*^21^ suggested that this disorder has relatively high heritability.

One of the first steps in QTL analyses is marker discovery. The genomics era has helped saturate genetic linkage maps, in comparison to a few years ago where maps would have a few hundred markers at most.^23^ GBS is one technique that can be used to generate thousands of markers for QTL mapping at relatively low cost.^35^ There remain some challenges, though, such as accurate genomic DNA quantification as variable DNA concentrations result in uneven sequence coverage across samples.^26^ In this study, there was a drastic difference in the number of reads generated for parent SPA440 compared with other parents: 11W-12-11 had nearly 5× as many reads, while Honeycrisp and Ambrosia individually had about 2.5× more reads. This may have been due to a lower DNA concentration or quality extracted from SPA440 samples compared to the other parents. Parental DNA samples were sequenced twice to ensure sufficient read counts for marker discovery, but this was still insufficient for the parent SPA440. Across samples, only 8% of reads were removed via the custom read filtering pipeline (Figure 1). However, nearly 40% of reads were discarded because they did not map to the reference genome. This is due in part to the fact that ~30% of the apple genome is absent from the anchored portion of the current reference genome sequence.^25^ When samples are distantly related to the reference genome, there can be problems with alignment and genotype calling.^4^ Read mapping will hopefully improve as the reference genome is improved over time, and the GBS reads generated here can be re-aligned to improved reference genome versions in the future and new SNPs can be called. Further drastic reductions in reads resulted from filtering SNPs for minor allele frequency, missing data and read depth using VCFtools, as well as marker selection for linkage map construction. After all steps were completed, only ~2% of the reads generated for this study were used for the final QTL analyses. Such reductions in usable reads have been observed in other work using GBS-derived SNP markers for consensus map construction in apple.^35^ Improvements to the apple reference genome, and imputation algorithms that help fill in missing data are needed to exploit the 98% of the data we did not make use of in this study.

Despite the fact that only 2% of sequence data were used for map construction, high-density genetic maps for two F_1_ crosses were generated. In a previous apple F_1_ cross, a consensus map was constructed with 1994 markers, totaling 1272 cM in length with an average of 0.68 cM between markers.^35^ The 11W-12-11×SPA440 and Ambrosia×Honeycrisp consensus maps in this study (Supplementary Figures S2 and S3, respectively) were composed of a similar number of markers (1918 and 2818, respectively), and had comparable average marker densities (0.83 cM and 0.52 cM, respectively), but were both larger (1586 cM and 1464 cM, respectively). Overall, both maps fall within the range of previously published map lengths.^43,44^ It is worth noting that 22% of the markers in both crosses had linkage group assignments that conflicted with the predicted chromosomal locations according to the reference genome. Such conflicts between genetic and physical positions have been reported previously; for example 18% of GBS-based SNPs in Gardner *et al.*^35^ and 14% of array-based SNPs in Antanaviciute *et al*.^45^ Incorrect anchoring of sequences in the reference genome assembly is the most likely explanation for this discrepancy and it is anticipated that future improvements to the reference genome will resolve most of these conflicts.

There is great interest in discovering the genetic basis of traits in apple due to the high costs associated with apple breeding and the potential of MAS to reduce these costs. Many QTL have been identified in apple, including loci underlying fruit size,^46,47^ weight,^47^ dry matter,^48^ ethylene production,^49^ firmness,^48^ volatile compounds,^50^ acidity,^51^ time of fruit maturity,^48^ fire blight resistance,^42^ bitter pit,^52^ skin russeting^53^ and rootstock-induced dwarfing.^54^ As of 2011, the majority of QTL studies involved several hundred markers and 250 individuals or fewer.^24^ The QTL analyses presented here used populations comparable in size, although larger populations (>500) are ideal.^48^ In the present study, two minor effect (<20% variation explained) QTL for scald were identified on chromosomes 2 and 3 in the parental background of 11W-12-11 (Figures 2 and 3), where the heterozygous states may increase the incidence of soft scald (Supplementary Figure S8). These QTL were associated with GO terms including enzymatic activity and binding (Supplementary Tables S1 and S2). It is noteworthy that the QTL peak on chromosome 2 in this study is in the vicinity of QTL associated with volatile compounds in other QTL mapping and genome wide association study (GWAS) work.^55,56^ In both studies, QTL at the beginning of chromosome 2 appear to be associated with various volatile compounds, including hexanol. Given the implication of compounds such as hexanol in the incidence of soft scald,^16,17^ these areas could prove relevant in elucidating the genetic basis of soft scald in the future. It is hoped that as annotation of the apple genome improves, further details about the genes adjacent to the QTL in this study will emerge. No other parental backgrounds revealed QTL (Supplementary Figures S4, S5, S6). One possible explanation for this could be that the low sample size of the Ambrosia×Honeycrisp cross (91 progeny in 2013) reduced power to identify QTL.

It is difficult to determine why the QTL identified in 2013 in 11W-12-11 were not repeated when analyzing 2014 data. Even in an established orchard, the amount of fruit produced by each genotype can vary across years, although the fluctuations we observed in 2013 versus 2014 were relatively drastic.^22^ In apple, the degree of stability of QTLs in diverse genetic backgrounds is largely unknown.^24,57^ In a QTL study of fruit quality, 1/3 of QTLs were stable over two harvest years, only one of which was a major QTL.^22^ Ideally, QTL results in apple would be compared between and within populations, across years and locations, but this is often prohibitively expensive and time consuming for apple.^22^ In the present study, it is likely that the lower sample size in 2014 resulted in a lack of power. Determining the stability and transferability of the QTL discovered here will clearly require studies with larger sample sizes and marker numbers. To this end, GWAS hold particular promise for identifying the causal loci underlying breeding targets in apple.^58^

One of the most important aspects of QTL analyses is phenotype data collection and interpretation. In apple mapping studies, it is particularly important to control for non-genetic variation, such as that imposed by the environment.^4^ For example, apple fruit texture is a complex trait that also shows considerable variation across cultivars, and efforts are being made to measure it comprehensively and efficiently.^59^ Another challenge to phenotyping in apple is studying how traits change during storage, and vary across years.^59^ In comparison to other disorders, little is known about soft scald,^12^ in part due to its unpredictable nature,^15^ with a range of susceptibilities across but also within cultivars.^8^ In this study, the correlation of disorder incidence across years was significant but low, which is highlighted by the difference in incidence between years in the susceptible parents 11W-12-11 and Honeycrisp (Supplementary Figure S1). Fruit maturity might affect soft scald development,^14^ which is a concern as there is currently no universally accepted and non-destructive method of measuring apple maturity,^59^ although the DA meter appears promising.^31–33^ Even for a cultivar like Honeycrisp, authors have highlighted the lack of a significant harvest index, and suggested fruit be harvested based on color development.^9^ In addition, progeny of a cross do not reach maturity simultaneously, which complicates phenotyping considerably. For this study, because the optimal harvest dates of F_1_ offspring are always unknown, fruits were harvested based on a threshold of delta absorbance measured from the apple skin (*I*_AD_). A significant benefit of using the DA meter is that fruits are not lost due to destructive methods of measuring maturity. Methods of improving phenotyping efficiency and accuracy in apple are needed and should be explored.

In the present study, it is impossible to ascertain the reason for lack of repeatability for the observed QTL. On the basis of the information presented above, there clearly are interacting factors affecting manifestation of soft scald. Another likely limiting factor is the low sample size of Ambrosia×Honeycrisp in 2013, and low sample sizes for both populations in 2014. Thus, there are clearly significant challenges associated with accurately quantifying storage disorders in sufficient sample sizes for well-powered genetic mapping in apples, but the present study provides a motivating starting point.

Although a prime candidate for improvement through marker-assisted selection, advances in apple breeding have been met with challenges.^2^ One of the major limitations compared with other crops are the relatively high costs associated with maintaining large F_1_ populations for the many years needed to perform robust QTL mapping.^4^ There has been limited use of QTL in MAS due to problems such as stability across genetic backgrounds and environments.^22^ For these reasons, the use of well controlled and replicated GWAS populations holds particular promise for elucidating the genetic underpinnings of commercially important traits in apple.^56^ If soft scald is in fact controlled by many small effect loci, genomic selection could help overcome some limitations of standard QTL mapping.^24^ The present study demonstrates that, even though only a small fraction of the DNA sequence data were used for genetic mapping, GBS data from apple F_1_ populations produced genetic maps comparable in quality to other methods that are suitable for QTL analysis. As an added benefit, the NGS data collected here can be re-aligned to improved reference genomes and analyzed with better bioinformatics tools in the future, possibly revealing QTL missed in the present study. With accurate, reliable and replicated phenotype data from large populations, the contribution of NGS technologies to the improvement of long-lived perennials can be fully realized.
