# Supplementary figures and images for: QTL analysis of soft scald in two apple populations
Source: Hortic Res. 2016 Sep 14;3:16043–. doi: 10.1038/hortres.2016.43 (PMC5022660; doi:10.1038/hortres.2016.43)

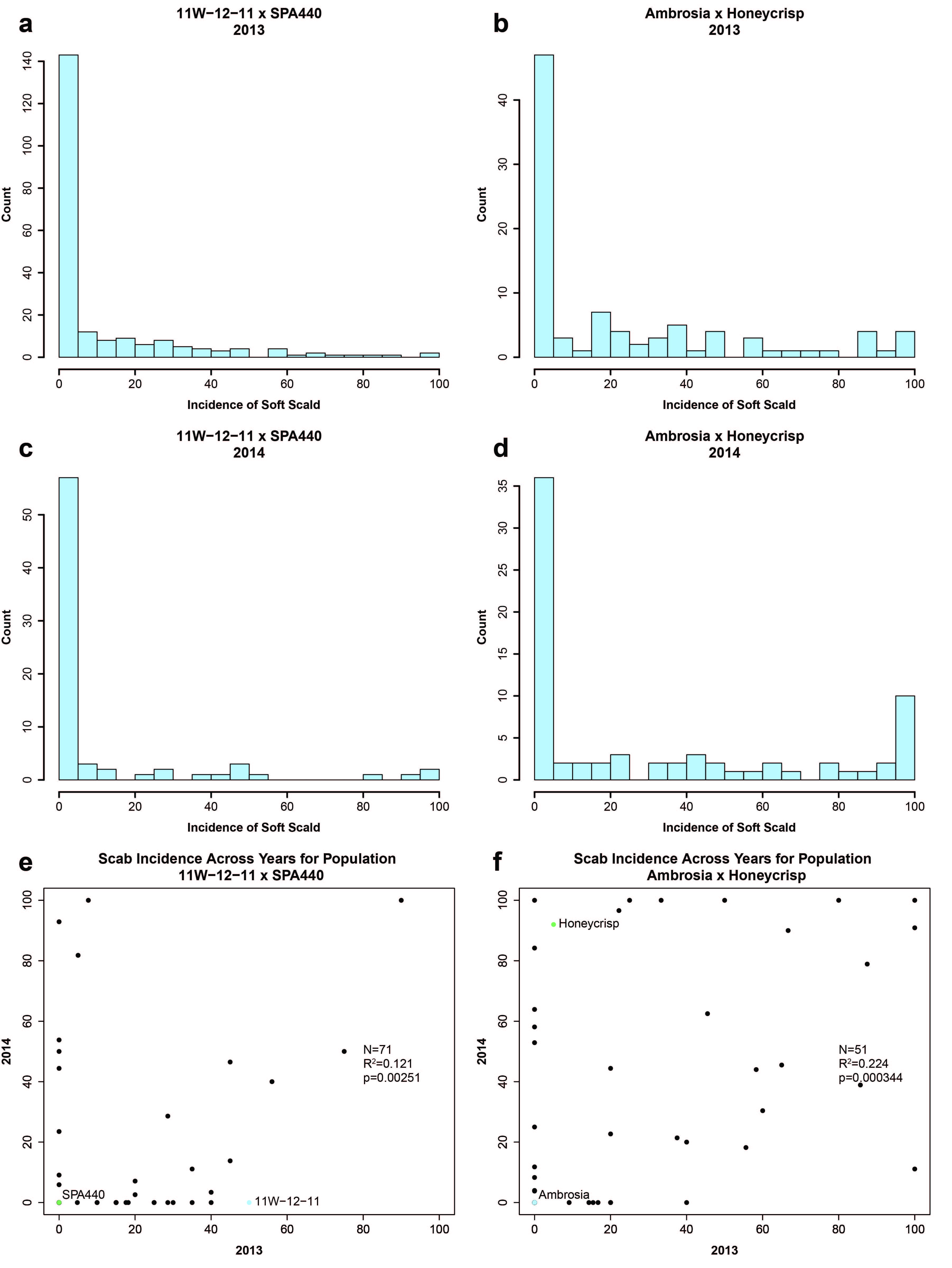

Supplement: Supplementary Figure S1 [file hortres201643-s4.jpg]

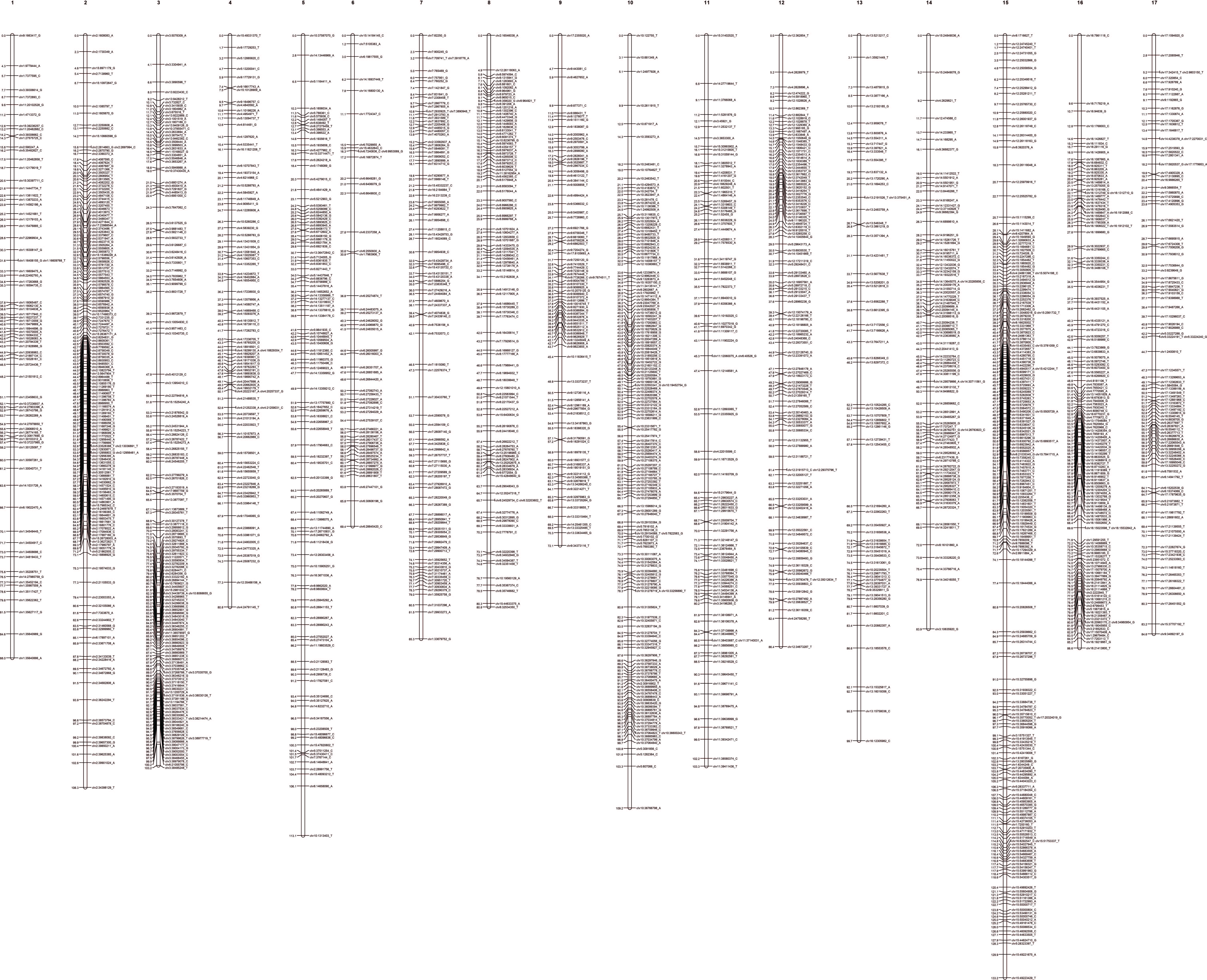

Supplement: Supplementary Figure S2 [file hortres201643-s5.jpg]

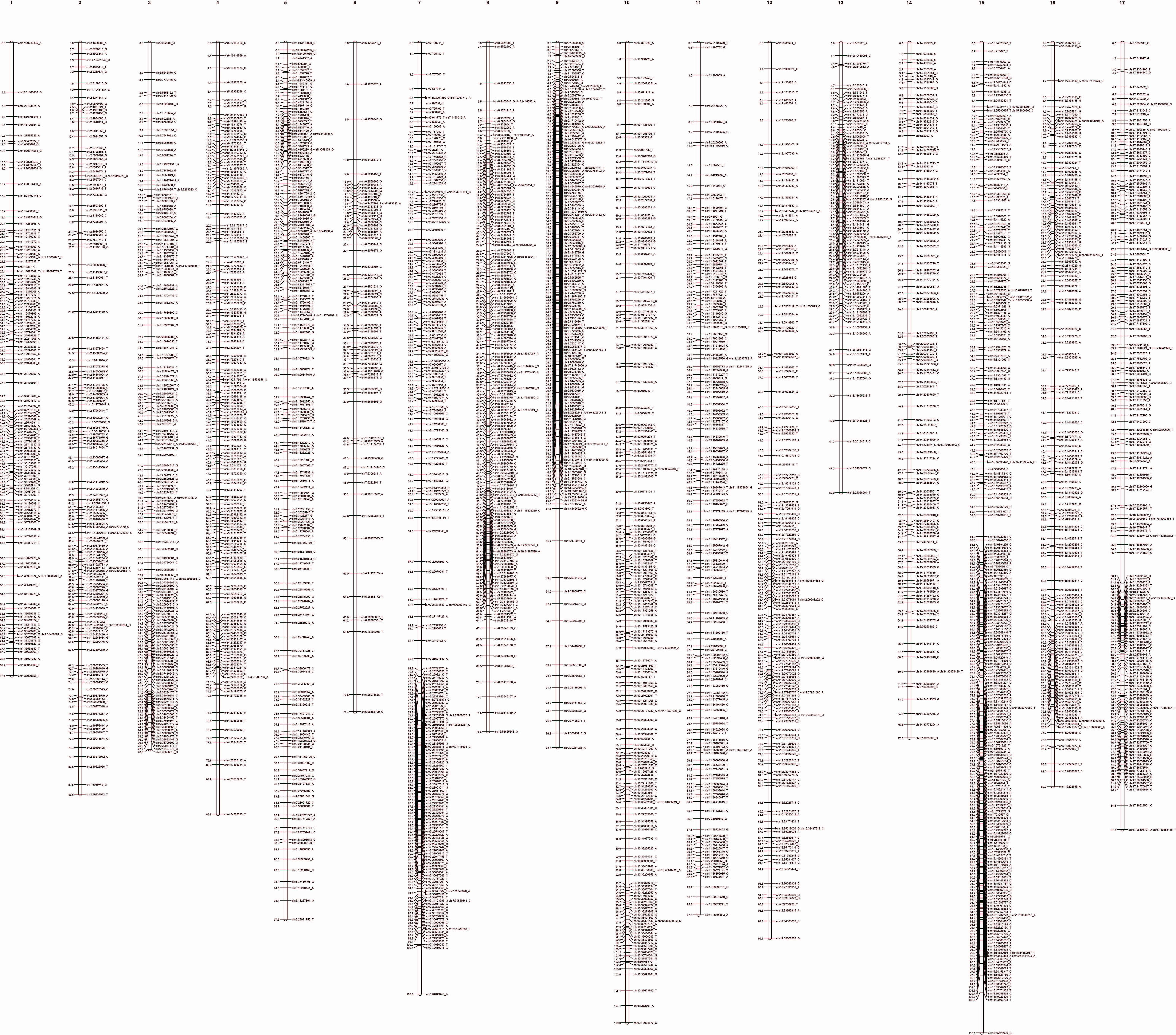

Supplement: Supplementary Figure S3 [file hortres201643-s6.jpg]

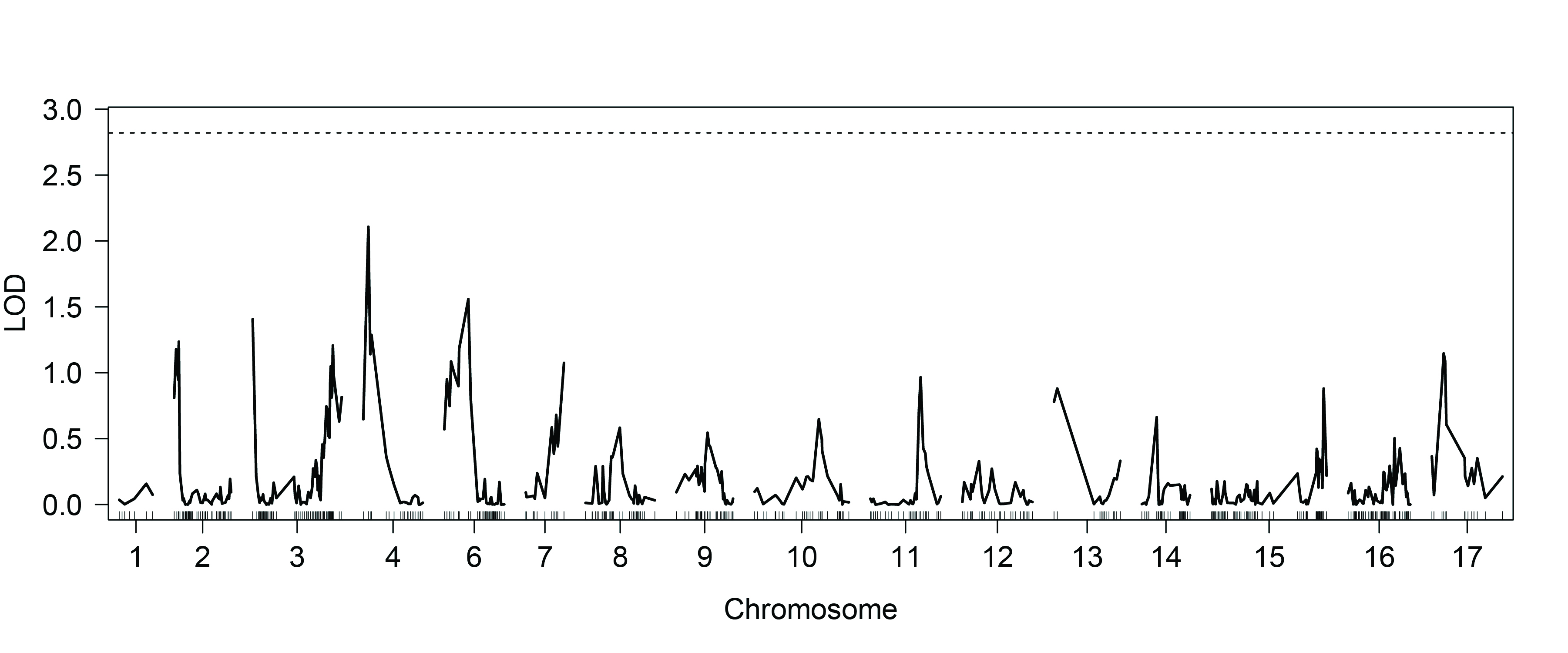

Supplement: Supplementary Figure S4 [file hortres201643-s7.jpg]

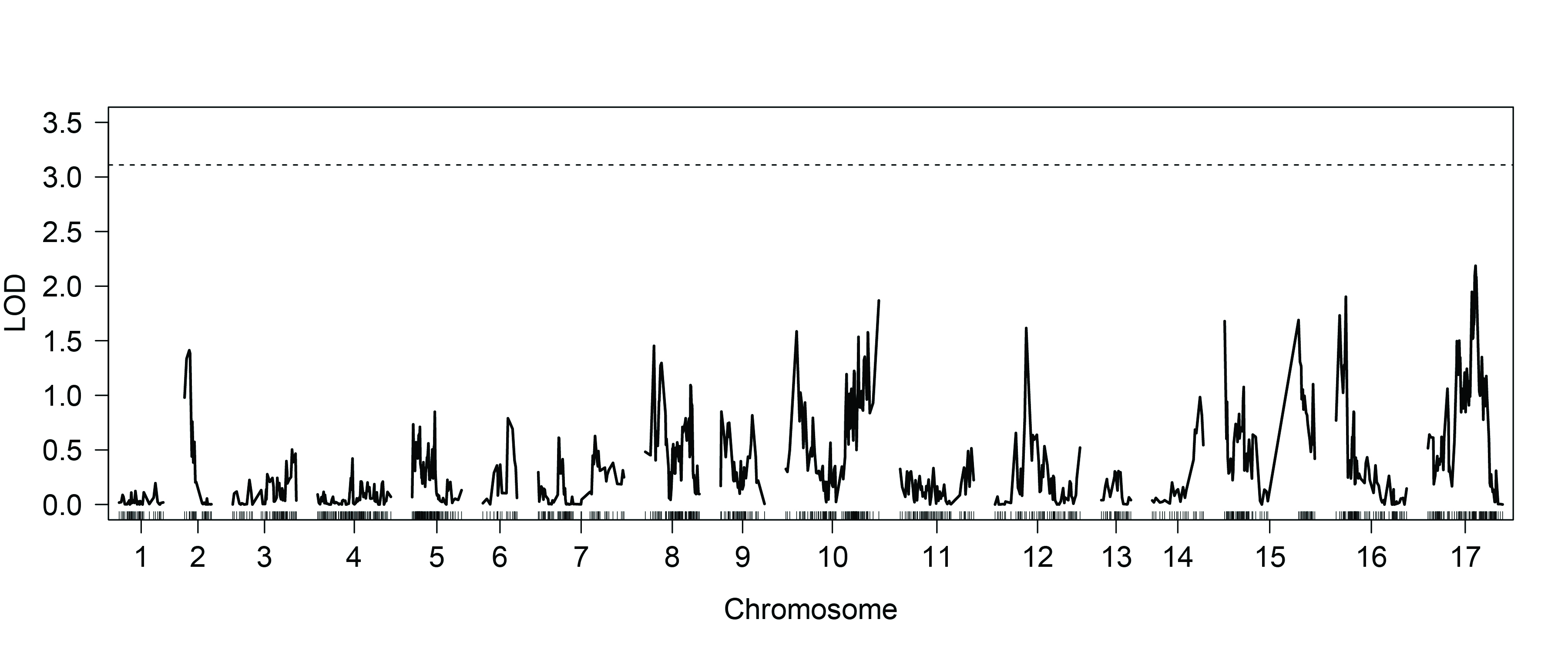

Supplement: Supplementary Figure S5 [file hortres201643-s8.jpg]

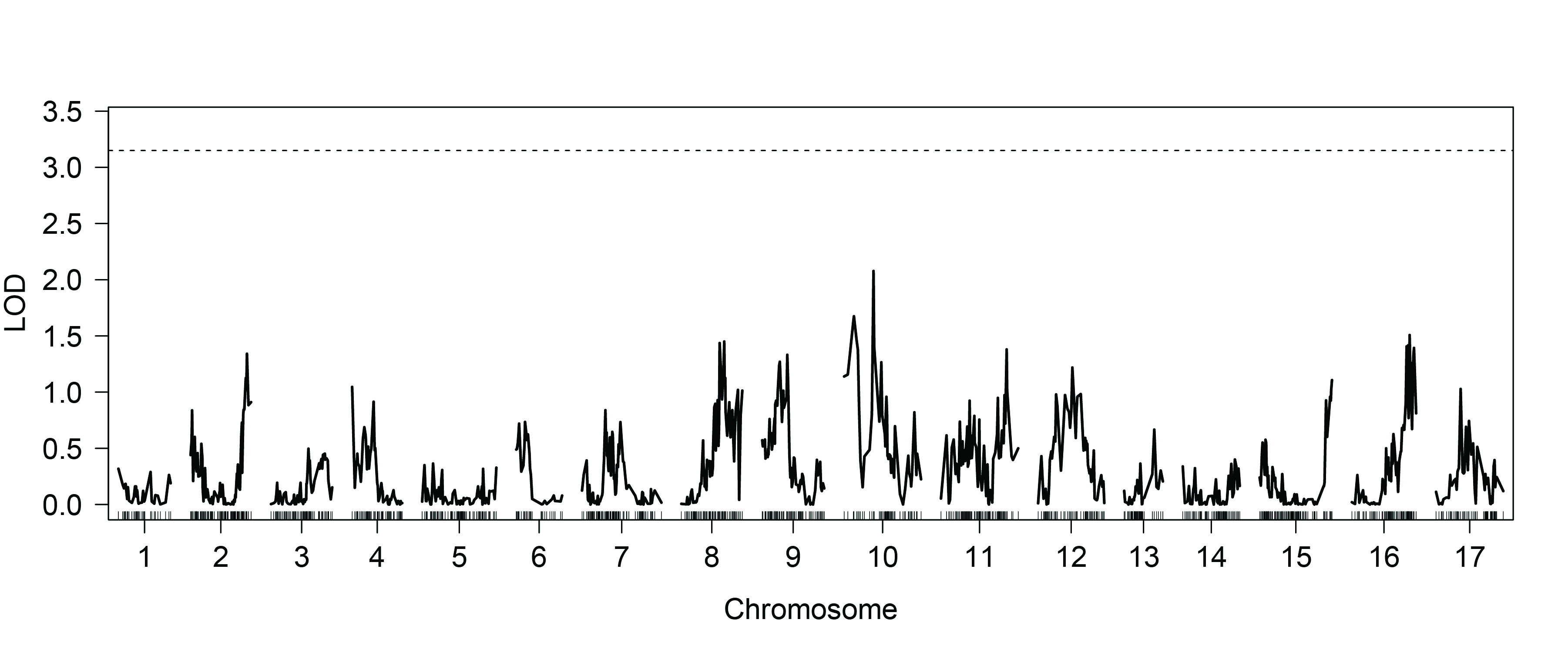

Supplement: Supplementary Figure S6 [file hortres201643-s9.jpg]

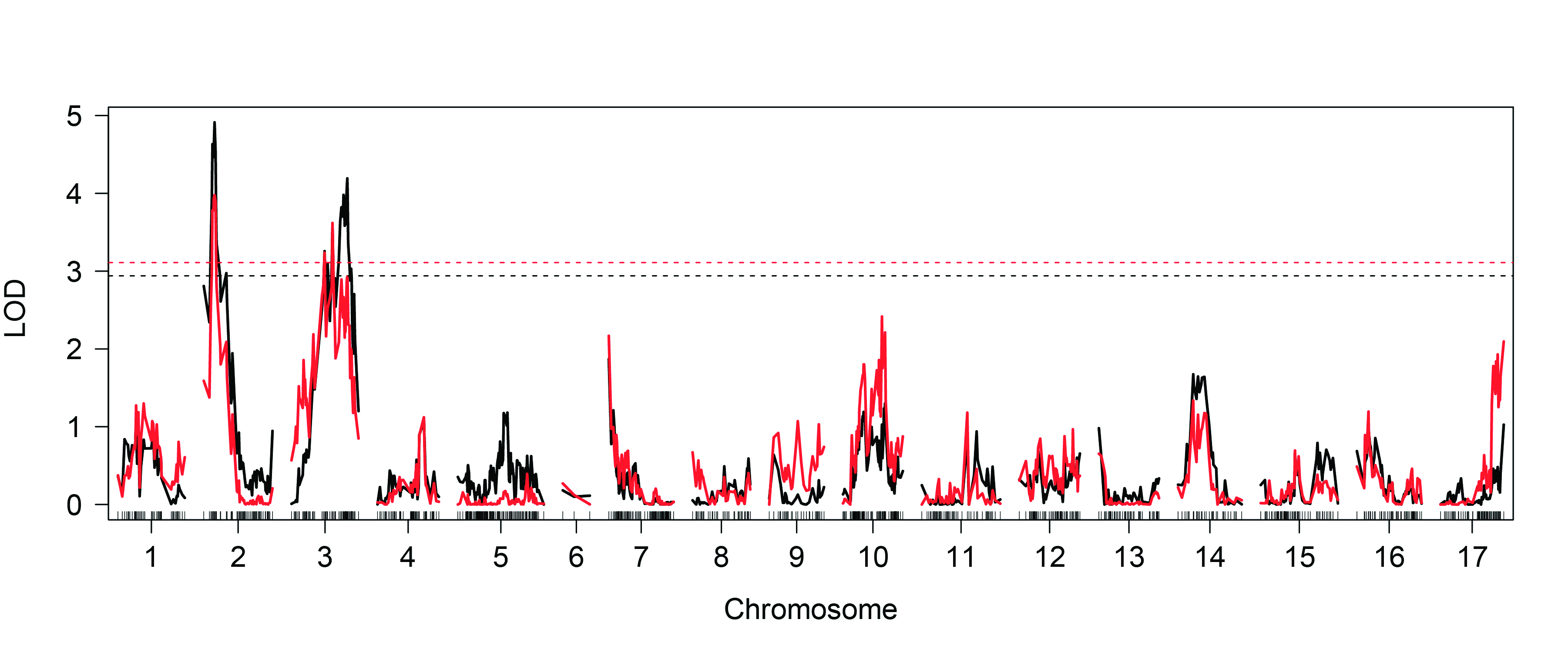

Supplement: Supplementary Figure S7 [file hortres201643-s10.jpg]

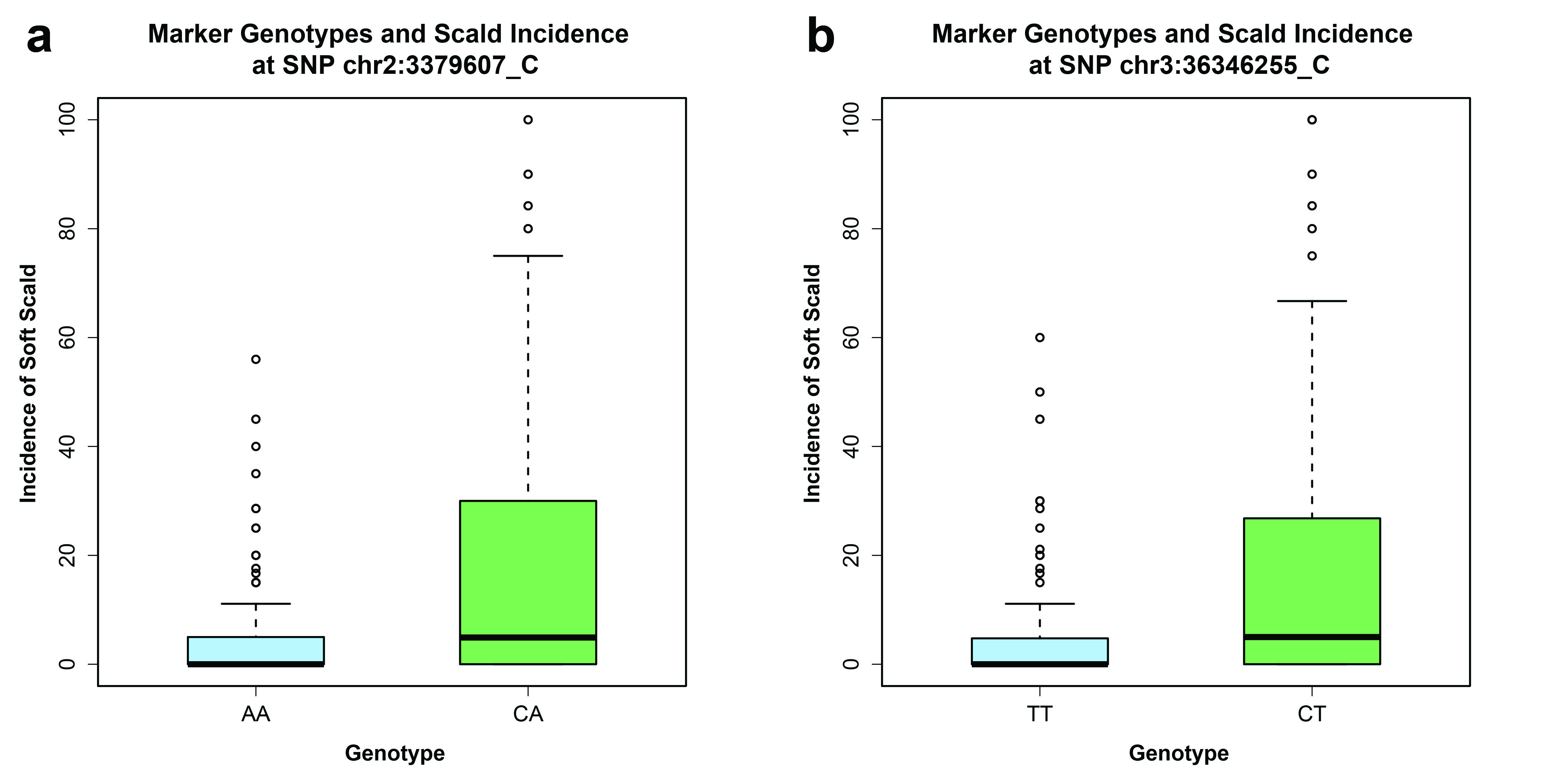

Supplement: Supplementary Figure S8 [file hortres201643-s11.jpg]
